# Mental health groups in high school students and later school dropout: a latent class and register-based follow-up analysis of the Danish National Youth Study

**DOI:** 10.1186/s40359-021-00621-7

**Published:** 2021-08-18

**Authors:** Susan Andersen, Michael Davidsen, Line Nielsen, Janne S. Tolstrup

**Affiliations:** National Institute of Public Health, University of Southern Denmark, Studiestræde 6, 1455 Copenhagen, Denmark

**Keywords:** Mental health, School dropout, High school students, Adolescence, Denmark, Latent class analysis

## Abstract

**Background:**

Mental health represents an important public health issue, and mental health problems have been linked to school dropout. This study aimed to identify mental health groups of high school students using both positive and negative aspects of mental health and to examine whether these mental health groups longitudinally predict school dropout.

**Methods:**

We conducted latent class analysis using the Danish National Youth Study 2014 (n = 60,526; mean 17.9 years) to identify clustering of mental health (11 items covering positive and negative aspects of emotional wellbeing and functioning in daily life), separately by sex. The relationship with subsequent school dropout was examined using logistic regression models, adjusted for age, ethnicity and socioeconomic status. Information on dropout status was obtained through educational registers.

**Results:**

School dropout rates was highest among first-year students. Four mental health groups were identified: Flourishing (females: 38%, males: 55%), moderate mental health (females: 15%, males: 20%), emotionally challenged (females: 28%, males: 15%) and languishing (females: 19%, males: 10%). Compared to the flourishing group, adjusted odds ratio (AOR) for dropout were 3.43 (95% CI: 2.98, 3.95), 1.73 (95% CI: 1.45, 2.06) and 1.76 (95% CI: 1.52, 2.04) in the languishing, moderate mentally healthy and emotional challenged females. Results in males were comparable.

**Conclusions:**

Mental health in high school students cluster together in four categories among both males and females. Students who are languishing, emotionally challenged or moderate mentally healthy have about 1.5-fold to threefold higher risk of dropping out of high school compared with flourishing students. Universal mental health interventions may be a promising strategy, particularly in the first year of high school where most students drop out of school.

**Supplementary Information:**

The online version contains supplementary material available at 10.1186/s40359-021-00621-7.

## Background

Youth is a developmentally crucial period for the onset of behaviours and conditions that shape future health and life conditions [1]. High school graduation constitutes an important life transition and necessary for future education and employment, and is, therefore, a major aim of governmental policies and interventions.

No or low education is linked to poor mental health [2] and is a strong determinant of morbidity and premature mortality [3, 4]. Education gives access to social support networks and improves job fit, financial literacy and health behaviours—all factors leading to higher life satisfaction [5]. Individuals who do not complete upper-secondary school are at higher risk of unemployment and low income [6] as well as increased health risk behaviour [2] than completers. It is more difficult for low-educated people to stay in the labour marked and poor physical and mental health followed by employment may further impact negatively on unemployment [7]. Several factors have been associated with high school dropout of which socioeconomic background and academic performance are some of the most well-documented determinants. An increasing number of studies have linked poor mental health [8, 9] and lack of social resources [10] with school dropout. For example, an Australian study showed that students with a prior distress disorder have twice the odds of high school dropout compared with those without [11].

Mental health is often conceptualized as having externalizing, internalizing or attention problems [12–14], or defined as mental disorders [15–18]. However, there is consensus that being mentally healthy is more than lack of problems and symptoms, and also comprises positive aspects such as general wellbeing, the perception of connectedness and ability to form positive relationships with others [19], high self-esteem, high self-efficacy [20] as well as overall good mood [21]. Positive mental wellbeing is a concept that often encompasses a hedonic dimension covering how people feel about themselves and their life and an eudemonic dimension which is the way people function in everyday life [22]. The eudemonic dimension mainly focuses on motivational and social aspects of functioning, although some studies define social wellbeing as a distinct component of mental health [23]. Ryan and Deci have conceptualized eudemonic wellbeing in their self-determination theory in which autonomy, competence and relatedness are seen as three necessities for personal growth, integrity and well-being [24]. Young people can also be categorized with different levels of mental health: flourishing, moderate or languishing mental health. Flourishing mental health refers to a combination of feeling good about and functioning well in life and languishing mental health is defined as not feeling good about and not functioning well in life [25].

It is difficult to capture mental health with few measurements [26]. Several mental health measures have been developed, for example the Warwick-Edinburgh Mental Well-being Scale [22]. However, these measures often necessitate a cut-off point on a continuous scale. This implies that individuals on either side of the threshold are very similar and heterogeneity within subgroups could be substantial [27]. Latent class analysis (LCA) is an explanatory method that is well-suited for investigation of underlying structures and may identify specific typologies (i.e., homogeneous subgroups) of mental health within populations. There may be certain patterns or combinations of mental health among high school students that generally co-occur.

Therefore, the aim of this study was two-fold. Firstly, we identify mental health characteristics of distinct subpopulations of high school students using both positive and negative aspects of mental health. Secondly, we examine the association between the identified mental health groups and later school dropout.

## Methods

### The Danish upper secondary education system

In Denmark, there is nine years of compulsory school (1st–9th grade) where lower secondary school refers to the 7–10th grade, with the 10th grade being optional. Afterwards, it is possible to enter upper secondary school for which Danish citizens (and many others meeting certain criteria) are offered state financial aid and are not charged tuition fees. There are two parallel opportunities: General upper secondary education and vocational education and training. General upper secondary education prepares students for higher education at tertiary level while vocational education and training programs provides the students with direct vocational qualifications leading to employment opportunities. Within general upper secondary education, there are four programs: Higher general examination (STX), higher preparatory examination (HF), higher commercial examination (HHX) and higher technical examination (HTX). These programs cover three years of education (students can enter after completing 9th grade), except HF that is a 2-year program but students must have completed 10th grade to enter. This study considers the STX and HF programs (16–19-year-olds), hereafter referred to as high school. Among students completing lower secondary school (i.e. 9th or 10th grade), about 80% are enrolled in upper secondary education (about 41% in STX and 8% in HF) [28].

### Study design

This follow-up study is performed among students in Danish high schools using data from the Danish National Youth Study 2014. The students who had answered the questionnaire survey were followed longitudinally in administrative registers at Statistics Denmark across an approximately four-year period until they achieved a high school diploma or dropped out of high school. The questionnaire was not designed specifically for this study. Permission was received to use the questionnaire.

### Participants

The Danish National Youth Study 2014 is a national survey conducted with the aim of investigating health and health behaviour among young people in upper-secondary education [29]. Data was collected from September 2014 to December 2014. All 137 high schools in Denmark were invited to participate, and 119 (87%) did participate. Of the invited students, 85% participated (n = 70,674). During school hours, students answered a web-based questionnaire, which consisted of 380 questions. Almost all participants (95%; n = 67,053) have been linked to their unique Personal Identification Number which enables linkage to national registers at Statistics Denmark. For this study, students not registered in the National Student Register during the questionnaire assessment period or with missing data on the mental health indicators (n = 6441) were excluded from analyses. Thus, the final study population consisted of 60,612 high school students.

### Measures

The applied data consists of a combination of survey data and register data.

#### Outcome: high school dropout

We defined dropout as the event of leaving high school without achieving a high school diploma. Students who switch to another institution or education program at the general upper secondary education level was not registered as dropout. It was based on data obtained from the Student Register at Statistic Denmark [30]. The Student Register is a cumulative register which is updated once a year (September 30), and information is obtained from the educational institutions' administrative register and goes through a comprehensive error check and validation of data [31]. The register contents education code for enrollment, code for graduation or dropout, start date and end date for each education at an individual level. Dates of dropout were used to identify dropouts within the follow-up period from autumn semester 2014 to 30^th^ September 2018.

#### Mental health indicators

The student questionnaire covered mental health indicators. Eleven dichotomous variables were used as indicators of mental health (each variable is described in Table 1). We included items that are reflecting both the hedonic or emotional wellbeing and the eudemonic wellbeing and functioning in daily life.

**Table 1:** Mental health items for the Class Analysis (LCA) divided into hedonic and eudemonic dimensions

	Items used as indicator	Response options	Coding to LCA
*Hedonic wellbeing:*			
Self-esteem	I am good enough the way I am	Strongly agree (1) Agree (2) Neither agree nor disagree (3) Disagree (4) Strongly disagree (5)	High Self-esteem (1–2) vs. Low or moderate self-esteem (3–5)
Life satisfaction	All things considered, how *satisfied* are you *with* your *life* as a whole these days	11-step ladder with ‘worst possible life’ = “0” and ‘best possible life’ = “10”	High life satisfaction (8–10) vs. Low or moderate life satisfaction (0–7)
Feeling low	How often have you experienced the following in the past six months: Feeling low	Almost every day (1) More than once a week (2) Almost every week (3) Almost every month (4) Rarely or never (5)	Weekly (1–3) vs. Less often than weekly (4–5)
Feeling irritable	How often have you experienced the following in the past six months: Irritable/bad tempered	Almost every day (1) More than once a week (2) Almost every week (3) Almost every month (4) Rarely or never (5)	Weekly (1–3) vs. Less often than weekly (4–5)
Feeling nervous	How often have you experienced the following in the past six months: Feeling nervous	Almost every day (1) More than once a week (2) Almost every week (3) Almost every month (4) Rarely or never (5)	Weekly (1–3) vs. Less often than weekly (4–5)
Stress	How often do you feel stressed?	Never/almost never’ (1) Monthly (2) Weekly (3) Daily (4)	Daily (4) vs. Not daily (1–3)
Loneliness	Do you feel lonely?	Yes, very often (1) Yes, often (2) Yes, sometimes (3) No (4)	Often lonely (1–2) vs. Not often lonely (3–4)
*Eudemonic wellbeing:*			
Autonomy	The past two weeks: I’ve been able to make up my own mind about things	None of the time (1) Rarely (2) Some of the time (3) Often (4) All of the time (5)	High autonomy (4–5) vs. Low or moderate autonomy (1–3)
Self-efficacy	How often can you manage the things you decide to do ?	Very often (1) Often (2) Sometimes (3) Rarely (4) Never (5)	High self-efficacy (1–2) vs. Low or moderate self-efficacy (3–5)
Social relatedness	How easy is it to talk to the following persons about things that really bother you? (your mother/father/friends)	Very easy (1) Easy (2) Difficult (3) Very difficult (4) Don’t have/see (5)	Easy (1–2) vs.Difficult/don’t have or see the person (3–5)

Life satisfaction defined as the degree to which a person evaluates the overall quality of his or her present life-as-a-whole positively [32] was measured using the Cantril ladder scale, which has been validated for use with a wide variety of populations, including adolescents [31]. Respondents were shown a ladder with steps ranging from 0 to 10 where 0 equals the worst possible life and 10 the best possible life. Their responses were dichotomized into high (8–10) versus low (0–7).

Self-esteem was measured by the statement ‘I am good enough the way I am’ with responses given on a 5-point Likert scale: Strongly agree (1), Agree (2), Neither agree nor disagree (3), Disagree (4), Strongly disagree (5). Responses were dichotomized into high self-esteem (1–2) versus low/moderate self-esteem (3–5).

Three items from the HBSC Symptom Check List [33] was used to measure negative affect/emotional symptoms. These were feeling low, feeling nervous and feeling irritable. The students were asked how often they have experienced these feelings during the past six months, which were dichotomized into weekly versus less than weekly.

Stress was measured by a single-item measure “How often do you feel stressed?”. Responses were dichotomized into ‘daily’ versus ‘weekly’, ‘monthly’, ‘never/hardly ever’.

Loneliness was assessed by a single item “How often do you feel lonely”. Responses were dichotomized into ‘very often’/’often’ versus ‘sometimes’/’no’.

Competence capacity to effectively act on the world and was captured by an item on self-efficacy: ‘How often can you manage the things you decide to do?’ and responses were dichotomized into ‘very often’/’often’ versus ‘sometimes’/’rarely’/’never’.

Autonomy defined as self-authorship or personal initiative was measure by an item from the Warwick-Edinburgh Mental Well-being Scale [22] with the statement: I’ve been able to make up my own mind about things’. The students were asked about their experience the past two weeks and their responses were dichotomized into ‘none of the time’/ ‘rarely’/ ‘some of the time’, versus ‘often’/ ‘all of the time’.

Social relatedness was measured by the item: “How easy is it to talk to the following persons about things that really bother you? (your mother/father/friends)”. Response options ranged from “very easy” to “very difficult”. We dichotomized the responses into trustful communication ‘(very easy’/’easy’) versus no trustful communication (‘very difficult’/’difficult’/ ‘neither difficult nor easy’) [34]. One variable for friends and one variable for parents were constructed. It was coded as trustful communication with parents was if the students had responded this to one of the parents.

#### Covariates

We used registers in Statistics Denmark [35] covering information on age (years), ethnicity and parental socioeconomic position (SEP) to be included as covariates. From the Danish Civil Registration System, we obtained information on age (years) and ethnicity measured by origin (determined by mother’s and father’s country of birth). Ethnicity was categorized as’Danish’, ‘descendants’ and ‘immigrants’. Indicator for SEP was taken from mother's and father's educational level. Information on parental education was retrieved from the Education Register and was defined as the highest achieved educational level of the parents. The educational levels were defined according to the seven-category International Standard Classification of Education (ISCED) as ‘Elementary school’ (ISCED levels 1–2), ‘Upper secondary education’ (ISCED level 3), ‘Short or medium tertiary education’ (ISCED levels 5–6) and ‘Long tertiary education’ (ISCED levels 7–8). ISCED level 4 is not present in the Danish educational system.

#### Statistical analysis

Latent class analysis (LCA) allows for the construction of underlying subgroups (classes) of mental health in our sample. Our latent class model was derived from the 11 indicators of mental health previously described. We fitted models by varying the number of classes between 2 and 7 using PROC LCA in SAS [36]. For each model, we assessed the goodness of fit using the likelihood ratio chi-square test (G-squared), the Akaike information criteria (AIC) and Bayesian Information Criteria (BIC). Lower values indicate better fit and parsimony of the model. We selected the final model considering the model fit statistics and based on the interpretability of the results. After determining the final model, individuals’ posterior probabilities (i.e. the probabilities of each individual belonging to each of the classes) determined the class variable. We conducted logistic regression analysis in which the class variable was used as the exposure and dropout as the outcome. We calculated adjusted odds ratios (AOR) and 95% confidence intervals (CI) to examine how the mental health groups were related to dropout from high school. The models were adjusted for age, ethnicity, and parents’ educational level. The class with the highest probability of reported mental health in the LCA were used as reference. Missing data on confounder variables was minimal in the sample with most missing on parents’ educational level (1.0%). Given that females often report poorer mental health than males [37] and females are more likely to complete their education [38], we conducted sex-specific analyses. All models were implemented in SAS v9.4 (SAS Institute Inc., Cary, NC).

## Results

### Participant characteristics and dropout rates

The students’ sociodemographic background and the prevalence of the mental health indicators for females and males are presented in Table 2. The mean age was 18.0 ± 1.7 years for males and 17.8 ± 1.1 years for females. The majority was in three-year higher general examination (STX) high school program. Approximately one third of the students had parents with upper-secondary education. The prevalence of emotional wellbeing differed for males and females; with a larger proportion of females than males reporting emotional symptoms and a lower proportion reporting life satisfaction and self-esteem. Conversely, relatedness, self-efficacy and autonomy were more equally distributed across sexes.

**Table 2:** Baseline characteristics and mental health indicators among Danish high school students, 2014

	Males	Females
	(n = 23,180)	(n = 37,432)
Age, mean years (SD)	18.0 (1.2)	17.8 (1.1)
High school program, n (%)		
Higher general examination (STX)	21,412 (92)	34,681 (93)
Higher preparatory examination (HF)	1768 (7.6)	2751 (7.4)
Ethnicity, n (%)		
Danish	21,297 (92)	34,403 (92)
Descendant	1242 (5.4)	2161 (5.8)
Immigrant	615 (2.7)	828 (2.2)
Parents’ education, n (%)		
Elementary school	776 (3.4)	1682 (4.5)
Upper-secondary	7090 (31)	13,686 (37)
Short or medium tertiary education	9095 (40)	14,402 (39)
Long tertiary education	5978 (26)	7265 (20)
*Mental health indicators:*		
High life satisfaction, n (%)	12,824 (55)	15,654 (42)
High self-esteem, n (%)	19,255 (83)	24,247 (65)
Daily stress, n (%)	1575 (6.8)	5654 (15)
Nervous, n (%)	52,567 (23)	13,640 (36)
Sadness, n (%)	3363 (15)	14,798 (40)
Irritable, n (%)	7805 (34)	20,006 (53)
Loneliness, n (%)	1383 (6.0)	4197 (11)
Relatedness to parents, n (%)	18,293 (79)	30,185 (81)
Relatedness to friends, n (%)	18,591 (80)	30,475 (81)
High self-efficacy, n (%)	20,246 (87)	29,841 (80)
High autonomy, n (%)	20,705 (89)	31,335 (84)

School dropout rates differed by sex, high school program and year of high school (see Additional file: Table S1), with the highest dropout rate in male first-year students (21% and 10%, respectively, for higher preparation (HF) and higher general examination (STX) programs). The dropout rates of second- and third-year students were lower (ranging from 1.4 to 6.7%).

### Mental health subgroups

**Fig. 1:**
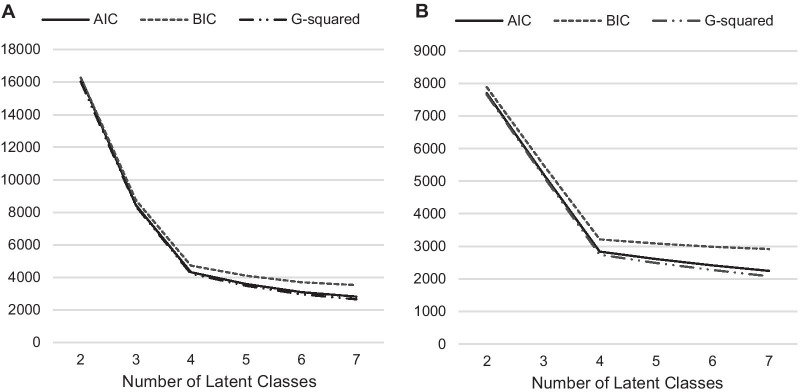
Model fit for latent class analysis models. A = Females; B = Males. AIC = Akaike´s information criterion; BIC = Bayesian information criterion

Results from the LCA suggested that a four‐class solution was the best fit for both females and males (Fig. 1). Although the AIC and BIC values continued to decrease as more latent classes were added to the model, the four-class model was selected because the drop in BIC and AIC for successive models was much less substantial after four classes. The four-class model also appeared to have distinguishable classes that were not trivial in size. The four-class LCA model parameters, including class membership probabilities and item response probabilities, are presented in Table 3.

**Table 3:** The expected proportions of the high school population in each class and item-response probabilities, by sex

	Class 1: Flourishing	Class 2: Moderate mentally healthy	Class 3: Emotionally challenged	Class 4: Languishing
	%	%	%	%
*Males*				
Latent class prevalence	55	20	15	10
Positive indicators				
High life satisfaction	78	25	46	4
High self-esteem	97	66	87	30
Relatedness to parents	91	61	80	46
Relatedness to friends	92	62	84	45
High self-efficacy	96	75	90	57
Autonomy	95	78	93	74
Negative indicators				
Stress	2	07	11	30
Nervous	6	15	62	75
Sadness	0.03	3	38	81
Irritable	10	24	93	95
Loneliness	0.01	7	3	41
*Females*				
Latent class prevalence	38	15	28	19
Positive indicators				
High life satisfaction	74	21	35	2
High self-esteem	92	47	69	16
Relatedness to parents	94	71	85	53
Relatedness to friends	96	75	87	49
High self-efficacy	95	71	85	48
Autonomy	94	74	89	62
Negative indicators				
Stress	3	10	18	39
Nervous	06	15	59	80
Sadness	1	10	69	96
Irritable	14	19	95	97
Loneliness	0.01	08	5	46

The largest class (females: 38%, males: 55%) scored high on all positive mental health indicators and had low probabilities of frequent occurring emotional symptoms when compared with all other classes. This was labelled ‘Flourishing’. Class 2 was labelled ‘Moderate mentally healthy’ (females: 15%, males: 20%) because it was characterized by students who scored relatively low on both feeling and functioning dimensions, for example they had reduced probabilities of life satisfaction (females: 21%, males: 25%) and self-esteem (females: 47%, males: 66%). However, their scores on symptoms were only slightly elevated. For example, 15% of males and females reported nervousness compared to 6% in the flourishing group and around 60%-80% in the two other groups. Class 3 consisted of an “Emotionally challenged” group (females: 28%, males: 15%) and emerged as students who had regular emotional symptoms but otherwise scored relatively high on the positive mental health indicators. Particularly, students in this class reported elevated probabilities of irritability (females: 95%, males: 93%), nervousness (females: 59%, males: 62%) and seven out of ten females reported sadness on a weekly basis. Class 4 was labelled ‘Languishing’ (females: 19%, males: 10%) which refers to low probabilities of positive mental health and high probabilities of negative mental health across all indicators, compared with the other three classes. For example, the probability of high life satisfaction was less than 5%, about one third of the students reported weekly stress (females: 39%, males: 30%) and the students had very elevated probabilities of loneliness (females: 46%, males. 41%) and feeling sad (females: 96%, males. 81%).

### Mental health and school dropout

School dropout was lowest in the flourishing group (males: 5.0%; females: 2.4%) and highest in the languishing group (males: 12.1%; females: 7.8%) (Table 4). The languishing group had about three times higher odds of dropping out of school than students from the flourishing group (males: adjusted odds ratio (AOR) = 2.70, 95% CI: 2.32 to 3.14; females: AOR = 3.43, 95% CI 2.98 to 3.95). Students from the moderate mental health group and the emotionally challenged group had also higher odds of dropping out compared to the flourishing group. In the moderate mental health group, males had an AOR of 1.43 (95% CI: 1.24 to 1.66) of dropping out of school and females had an AOR of 1.73 (95% CI: 1.45 to 2.06). In the emotionally challenged group, both males and females had 1.6-fold or more increased odds of school dropout (males: AOR = 1.60, 95% CI 1.37 to 1.88; females: AOR = 1.76, 95% CI 1.52 to 12.04). When accounting for high school program (i.e. the two-year HF vs. the three-year STX), all analyses produced similar results (Additional file 1: Table S2).

**Table 4:** Odds ratios for dropping out of school by mental health groups among Danish high school students

	Dropout	Unadjusted		Adjusted by age, ethnicity and parents’ education	
	%	OR	95% CI	AOR	95% CI
*Males*					
Flourishing (ref.)	5.0	1.00	–	1.00	–
Moderate mentally healthy	7.1	1.45	1.26 to 1.67	1.43	1.24 to 1.66
Emotionally challenged	7.9	1.62	1.39 to 1.89	1.60	1.37 to 1.88
Languishing	12.1	2.62	2.26 to 3.04	2.70	2.32 to 3.14
*Females*					
Flourishing (ref.)	2.4	1.00		1.00	
Moderate mentally healthy	4.3	1.81	1.53 to 2.15	1.73	1.45 to 2.06
Emotionally challenged	4.1	1.69	1.46 to 1.95	1.76	1.52 to 2.04
Languishing	7.8	3.37	2.94 to 3.87	3.43	2.98 to 3.95

OR = Odds Ratio; AOR = Adjusted Odds Ratio; CI = Confidence Interval. For all models: *p* value < 0.0001

Restricting the analysis to those who were in the first year of high school produced similar odds ratios for the association between mental health and school dropout (somewhat stronger for males and slightly lower for females; see Table 5). For males, AORs ranged from 1.58 (95% CI: 1.31 to 1.92) to 2.89 (95% CI: 2.35 to 3.55). For females, AORs ranged from 1.49 (95% CI: 1.21 to 1.90) to 3.29 (95% CI: 2.79 to 3.98).

**Table 5:** Odds ratios for dropping out of the first year of high school by mental health groups among Danish high school students

	Dropout	Adjusted by ethnicity and parents’ education	
	%	AOR	95% CI
*Males* (n = 8498)			
Flourishing (ref.)	8.4	1.00	–
Moderate mentally healthy	13.3	1.58	1.31 to 1.92
Emotionally challenged	13.5	1.71	1.41 to 2.08
Languishing	21.0	2.89	2.35 to 3.55
*Females* (n = 13,762)			
Flourishing (ref.)	4.4	1.00	–
Moderate mentally healthy	6.9	1.49	1.21 to 1.90
Emotionally challenged	7.3	1.73	1.45 to 2.08
Languishing	13.3	3.29	2.79 to 3.98

AOR = odds ratio, CI = confidence interval. For all models: *p* value < 0.0001

## Discussion

This study adds to the growing literature on the complex associations between mental health and dropout in high school youth. We used Latent Class Analysis (LCA) and identified four classes of mental health among Danish high school students: Flourishing, moderate mental health, emotionally challenged and languishing. Compared to the flourishing group, those with lowest mental health—the languishing group—had the highest odds of school dropout. However, the analysis showed that both the moderate mentally healthy and emotionally challenged groups also had higher risks of dropping out compared to the flourishing group. In addition, our analysis uncovered that most students drop out of high school in the first year of school; nevertheless, the association between mental health and dropout was somewhat similar to the total student population.

Our results resemble other findings. For example, Suldo et al. [39] identified four groups labelled complete mental health, vulnerable, symptomatic but content, and troubled among US high school students. Despite a lower age range (mean 15 years versus 18 years in our sample), these groups correspond to the identified mental health groups in our study. For example, the sizes of the lowest mentally healthy groups seem similar; Suldo et al. [39] found that 15% was troubled and we found that 10% of males and 19% of females were languishing. A Danish study of 16–29-year-olds identified that 29% women and 19% men had poor mental health [8]. The latter study did not find an association between mental health and high school dropout, but the measurement of mental health was based on the 12-item Short-Form Health Survey and dichotomized into good versus poor mental health, which may have resulted in substantial heterogeneity within the two subgroups and subsequent attenuated association [27]. Many studies indicate that externalizing problems among students are associated with dropout from upper-secondary schools. For example, a study by Sagatun et al. [40] suggests that externalizing problems increased dropout for both males and females. In contrast, for internalizing problem, dropout was only increased for females [40]. Nevertheless, we found that that internalizing factors (i.e. emotional problems such as sadness and nervousness) were associated with increased dropout for both males and females. The mechanism between poor mental health and dropout may be explained by loss of concentration and truant behavior [15]. Another possibility is reverse causality, i.e. that schoolwork-related problems may adversely affect mental health.

The merits of this study are (1) the longitudinal design with combination of questionnaire- and register-based data which precluded attrition bias, (2) the large study population and high participation rate, (3) high validity and reliability of the outcome measure and (4) the use of several indicators of positive and negative mental health reflecting both hedonic and eudemonic wellbeing. Particularly, the longitudinal design is a strength as compared to prior research within the area, where the use of retrospective data has been a major limitation [41]. There are also some methodological limitations. First, the risk of residual confounding must be considered. The present study goes some way towards ruling out earlier‐proposed environmental drivers, such as family socio‐economic disadvantage, because we adjusted for the educational level of parents. However, adverse childhood experiences and learning difficulties are possible confounding factors that are likely play a role in achieving a high school diploma [42] and affects mental health as well. Second, we were dependent on selecting the mental health indicators from a survey that was initially developed for other purposes. The Danish National Youth Study 2014 is a population-based study that monitored risk factors, behaviour and health of high school youth across multiple domains and aims to contribute to a wide knowledge on adolescent health behaviour, health and well-being. Given the broad focus of the survey, detailed information about mental health such as externalizing problems was not available. This should be considered when interpreting our results. Third, despite the study was based on a nationally representative high school sample, the results might not generalize to other school settings because the school environment, programs and curricula targeted at preventing dropout and promote mental health may differ. Finally, many factors may contribute to students dropping out from high school, for example lack of motivation and significant life events [43]. In this study, our interest was in mental health during the high school time, but the contribution of various factors and their interaction effect can be a focus for future research.

### Implications

Segmenting the high school population into specific groups based on a range of mental health indicators may improve the scope, utilization and efficacy of interventions that target dropout issues in high school. Our study showed that not only the group with languishing mental health but also the moderate mentally healthy and the emotionally challenged groups should be targeted in interventions promoting mental health in order to prevent dropout. This is particularly relevant in the first year of high school, which is the period with the highest level of dropout. Because these three mental groups constitute a large part of the high school population (about 45% of males and 62% of females), the interventions should be universal and designed to impact all students. Recent reviews on school mental health promotion have shown some success for universal interventions to promote mental health among students [44, 45]. Mental health promotion in schools needs to be achieved through the provision of a continuum of intervention components focusing on social and emotional learning, competence for all students, and actively involve young people, schools and communities [44]. In addition, there seems to be a potential in integrating digital interventions in school mental health promotion [46].

## Conclusions

This study identified four distinct mental health groups using LCA in a Danish nationally representative high school sample. Our study showed that students who are moderate mentally health, have frequent occurring emotional symptoms or experience languishing mental health have about 1.5-fold to threefold higher risk of dropping out of high school compared with flourishing students. As the three mental health groups constitute around half of the high school population, universal interventions designed to impact all students may be a promising strategy, particularly in the first year of high school where most students drop out of school.

## Supplementary Information

**Additional file 1**.** Table S1**. School dropout rates among Danish high school students, by high school program and year.** Table S2**. Odds ratios for dropping out of high school by mental health groups among Danish high school students, adjusted by age, ethnicity, parents’ education and high school program.

## Data Availability

The datasets generated during the current study are not publicly available due to data being stored by Statistics Denmark. The authors cannot share or make the dataset publicly available because it is illegal to export individual level data. Interested readers or researchers must request Statistics Denmark (www.dst.dk) and contact the corresponding authors of this study.

## Abbreviations

AOR: Adjusted odds ratio
CI: Confidence interval
ISCED: International Standard Classification of Education
LCA: Latent class analysis
N: Number
