# Supplementary material for: Mental health groups in high school students and later school dropout: a latent class and register-based follow-up analysis of the Danish National Youth Study
Source: BMC Psychol. 2021 Aug 18;9:122. doi: 10.1186/s40359-021-00621-7 (PMC8371858; doi:10.1186/s40359-021-00621-7)
Supplement: Supplementary file 1 — Additional file 1. Table S1. School dropout rates among Danish high school students, by high school program and year. Table S2. Odds ratios for dropping out of high school by mental health groups among Danish high school students, adjusted by age, ethnicity, parents’ education and high school program. [file 40359_2021_621_MOESM1_ESM.docx]

**Table S1** School dropout rates among Danish high school students, by high school program and year

|  | **Males**  **Boys** | | **Females**  **Girls** | |
| --- | --- | --- | --- | --- |
|  | N | % | N | % |
| Higher general examination (STX) |  |  |  |  |
| First-year student | 750/7,605 | 9.9 | 812/12,364 | 6.6 |
| Second-year student | 364/7,292 | 5.0 | 339/11,617 | 2.9 |
| Third-year student | 129/6,481 | 2.0 | 148/10,605 | 1.4 |
| Higher preparatory examination (HF) |  |  |  |  |
| First-year student | 183/893 | 20.5 | 174/1,397 | 12.5 |
| Second-year student | 56/838 | 6.7 | 70/1,329 | 5.3 |

**Table S2** Odds ratios for dropping out of high school by mental health groups among Danish high school students, adjusted by age, ethnicity, parents’ education and high school program

|  | **Males** | |  | **Females** | |
| --- | --- | --- | --- | --- | --- |
|  | **AOR** | **95% CI** |  | **AOR** | **95% CI** |
| Mental health groups |  |  |  |  |  |
| Flourishing (ref.) | 1.00 | - |  | 1.00 | - |
| Moderate mentally healthy | 1.44 | 1.24 to 1.66 |  | 1.70 | 1.43 to 2.03 |
| Emotionally challenged | 1.58 | 1.34 to 1.85 |  | 1.77 | 1.53 to 2.04 |
| Languishing | 2.73 | 2.34 to 3.17 |  | 3.38 | 2.93 to 3.85 |
| Age, years | 0.80 | 0.76 to 0.84 |  | 0.80 | 0.76 to 0.84 |
| Ethnicity |  |  |  |  |  |
| Danish (ref.) | 1.00 | - |  | 1.00 | - |
| Descendant | 1.69 | 1.39 to 2.04 |  | 1.69 | 1.41 to 2.02 |
| Immigrant | 2.23 | 1.72 to 2.90 |  | 2.62 | 2.27 to 3.08 |
| Parents’ education |  |  |  |  |  |
| Elementary school | 2.93 | 2.17 to 3.79 |  | 2.32 | 1.85 to 2.91 |
| Upper-secondary | 2.10 | 1.79 to 2.46 |  | 1.46 | 1.25 to 1.72 |
| Short or medium tertiary education | 1.45 | 1.24 to 1.71 |  | 1.12 | 0.95 to 1.32 |
| Long tertiary education (ref.) | 1.00 | - |  | 1.00 | - |
| High school program |  |  |  |  |  |
| Higher general examination (ref.) | 1.00 | - |  | 1.00 | - |
| Higher preparatory examination | 2.80 | 1.38 to 3.30 |  | 2.64 | 2.27 to 3.08 |

AOR = Odds Ratio; CI = Confidence Interval. For all models: *p* value < 0.0001
